# Influence of the pili of *Lacticaseibacillus rhamnosus* GG on its encapsulation and survival in mixed protein-starch gels assembled by *in situ* fermentation

**DOI:** 10.1128/aem.00248-25

**Published:** 2025-06-12

**Authors:** Tana Hernandez-Barrueta, Silvia Lorena Amaya-Llano, Nitin Nitin

**Affiliations:** 1 Department of Food Science and Technology, University of California 117239, Davis, California, USA; 2 Posgrado de Alimentos, Universidad Autonoma de Queretaro 27772, Santiago de Queretaro, Queretaro, Mexico; 3 Department of Biological and Agricultural Engineering, University of California 240434, Davis, California, USA; Universita degli Studi di Napoli Federico II, Portici, Italy

**Keywords:** encapsulation, probiotic, pili, gel, protein, starch

## Abstract

Preserving the viability of probiotics during storage and gastrointestinal digestion poses a significant challenge in the development of effective probiotic formulations. Thus, this study developed an *in situ* fermentation approach to encapsulate the probiotic *Lacticaseibacillus rhamnosus* GG (LGG) in a mixed whey protein/modified starch gel and evaluated (i) the role of the pili on gel formation and on the effectiveness of the gel to maintain cell viability during simulated digestion, and (ii) the storage stability of the encapsulated probiotics. Kinetic data of gels made with the wild-type (WT) or a pilus-depleted mutant (Δ*spaCBA*) strain showed a rapid *in situ* gel formation (<30 min) at room temperature after inoculating the polymeric mixture, driven by *in situ* fermentation and independently of the piliation of the cells. After simulated gastrointestinal digestion, the viability of encapsulated WT cells was ~3 log higher than free WT cells (*P* value 7 × 10^−4^) and ~0.6 times higher than encapsulated Δ*spaCBA* cells (*P* value 9 × 10^−3^). A higher release of Δ*spaCBA* vs WT cells from the gels was quantified, and confocal microscopy revealed the aggregation of Δ*spaCBA* but not WT cells within the gel cavities. These findings suggest the pili-dependent retention of LGG within the gel contributes to its protective effect. Finally, the hydrated gels sustained counts of LGG of 7.76–6.69 log CFU/g (depending on the relative humidity) during 2 months of storage at room temperature. In summary, bacteria-to-matrix interactions might influence the survival of probiotics during delivery, and the protein/starch gels could represent a cost-effective alternative for unrefrigerated storage and delivery of probiotics.

**IMPORTANCE:**

Many probiotic formulations struggle to maintain the viability of microbial cells over time and during their passage through the gastrointestinal tract. This has led to the development of encapsulation strategies for probiotics, most of which are either costly to implement or damage the cells during the encapsulation process. To overcome these limitations, this work developed a rapid fermentation-based approach to encapsulate probiotics in protein/starch gels as a strategy to keep the cells alive during storage and digestion. Moreover, this work explored the role of interactions between bacterial cells and their encapsulation matrix on the formation of the gels and in the protection the gels provided in maintaining the viability of cells during simulated digestion. Developing this *in situ* fermentation approach for the encapsulation of probiotics and understanding the bacteria–matrix interactions will lead to the development of more effective probiotic products that can be easily deployed in low-resource settings.

## INTRODUCTION

Growing evidence suggests that probiotics could be key in addressing critical global health issues, such as infant malnutrition, a condition that disproportionately impacts low- and middle-income countries (1). For example, administering *Lacticaseibacillus rhamnosus* GG (LGG) to children suffering from severe acute malnutrition (alone or in combination with other probiotics) has been shown to reduce the incidence and duration of diarrhea (2–5), which is the third leading cause of death for children under five worldwide (6). The efficacy of such probiotic interventions depends on the viability of the cells, which must be preserved throughout manufacturing, distribution, storage, and digestion. This need has driven the development of encapsulation methods to enhance microbial stability and delivery. Common encapsulation approaches, such as spray drying, freeze drying, and extrusion, often expose cells to stressful conditions that may reduce their viability or require complex, energy-intensive processes and specialized equipment (7, 8) that limits their deployment in low-resource settings. Therefore, as we envision a broader role of probiotics in addressing global health challenges, it is essential to develop solutions that maintain cell viability during storage and digestion, while being practical to implement in a context of limited resources.

Among available encapsulation strategies, the self-assembly of oppositely charged polyelectrolytes could represent an energy-efficient process given its spontaneous nature. For instance, the layer-by-layer assembly technique involves sequentially coating cells with oppositely charged polymers that deposit on each other driven by electrostatic interactions (9). This technique has been used to encapsulate lactic acid bacteria in layers of zein and pectin, chitosan and sodium phytate, gelatin and green tea polysaccharides, etc., resulting in enhanced survival of the cells during storage, heating, and simulated digestive conditions (9–11). However, this method also requires multiple washing and centrifugation steps, which increase energy and water demands as the number of coating layers grows. Encapsulation through complex coacervation is another approach, wherein oppositely charged biopolymers attract and form a biopolymer-rich phase in which cells can be immobilized. Examples of this include the encapsulation of *L. rhamnosus* GG, *Lactobacillus acidophilus*, and *Bifidobacterium longum* in complex coacervates formed from mixtures of whey protein isolate and modified starch, pea protein isolate and sugar beet pectin, or gelatin and pectin (12–14). While coacervation offers high entrapment efficiency and minimal cell damage, it requires subsequent freeze or spray drying to achieve storage stability (12–14). Another promising approach involves the assembly of mixed protein-polysaccharide gels, which can form at room temperature and without agitation when conditions of pH, polymer concentration, and polymer ratio favor associative interactions between the polymers (15). Besides the low energetic requirements, the complexation between the protein and polysaccharides seems to slow down the degradation of the protein during simulated gastric conditions (16, 17), making these gels potential matrices suitable for the delivery of cargo to the gut. Gels of this kind have been structured*—*albeit not for the encapsulation of probiotics—using protein isolates from whey, casein, soy, amaranth, and quinoa combined with polysaccharides such as native and modified starches, xanthan gum, and carrageenan (18–25). Glucono-δ-lactone is commonly employed as an acidifying agent as it creates a gradual pH drop that resembles fermentation. Instead, the fermentative capacity of certain probiotic strains could be exploited. Compared to the other encapsulation methods discussed, fermentation has the advantage of being a process that can be easily deployed in low- and middle-income countries due to its simplicity and familiarity (26).

In this work, we took advantage of the acidifying capabilities of the probiotic *L. rhamnosus* GG to encapsulate these cells in a mixed protein-polysaccharide gel assembled by *in situ* fermentation, as an approach to stabilize the microbes during simulated digestion and storage. As the polysaccharide, we selected a starch modified by acid hydrolysis and extrusion, which, apart from serving as a structural component of the gel, could provide *L. rhamnosus* GG with rapidly fermentable sugars such as glucose, which gets released from the starch during the modification process (27). As protein, we selected whey protein isolate. The rationale for choosing a combination of whey protein and modified starch was twofold: (i) these materials can interact electrostatically under acidic conditions, forming different gel-like structures depending on the total solids content, protein to starch ratio, and pH (13, 18, 28), and (ii) this mixture of biopolymers has been used to encapsulate lactic acid bacteria (e.g., by complex coacervation and/or spray drying) resulting in enhanced survival of the cells during digestion and storage (13, 27, 29). More importantly, whey protein was selected as it has been reported that *L. rhamnosus* GG can adhere to the proteins in whey. Although various classes of surface molecules have been predicted to participate in the binding of *L. rhamnosus* GG to β-lactoglobulin (e.g., mucin-binding protein and immunoglobulin-like fold) (30), experimental evidence has shown the key role that the pili play in such adhesion (30–33). The pili-mediated adhesion to milk proteins was correlated with an increased encapsulation efficiency of this probiotic strain in a dairy protein matrix (31). The pili of *L. rhamnosus* GG (known as the SpaCBA pili) are proteinaceous appendages that protrude outwardly from the surface of the cells, mediate the adhesion of the bacteria to mucin and epithelial/immune cells, and are involved in biofilm formation (34, 35). While there is *ex vivo* evidence of the cross-linking between the SpaCBA pili of *L. rhamnosus* GG and β-lactoglobulin (using purified pilus) (36), the influence of such interaction on *in situ* gel formation and subsequent cell release during digestion remains largely unexplored, despite their potential implications in the development of more efficient formulations for probiotic delivery. Thus, this study examined the effects of the SpaCBA pili on gel formation and cell protection during simulated digestion using a pilus-depleted mutant of *L. rhamnosus* GG.

To the best of our knowledge, this work is the first to evaluate the role that bacteria–matrix interactions have in the formation of gel-based encapsulation systems for probiotic delivery and the survival of the encapsulated cells during simulated digestion. The knowledge gained through this work might lead to the development of novel cost-effective strategies to encapsulate probiotics, based on the bacteria–matrix interactions during encapsulation and delivery of probiotics, and the use of minimal resources for the encapsulation process and storage of the product, which might accelerate the deployment of these technologies in low-resource settings.

## MATERIALS AND METHODS

### Materials

Whey protein isolate (9400 WPI) was donated by Hilmar Ingredients (Hilmar, CA, USA). According to the manufacturer, the protein, fat, ash, and moisture content (% in dry basis) was 90.06, 0.64, 2.68, and 4.22, respectively; lactose < 0.1%. Starch was extracted from huauzontle (*Chenopodium berlandierii* spp. *nuttallie*) seeds and modified by acid hydrolysis and extrusion as we previously reported (27). Rhodamine B (Cat No. 1001674965), bile salts (Cat No. 1002046452), tetracycline hydrochloride (Cat No. 1000791743), potassium phosphate monobasic (Cat No. 100250156), potassium acetate (CAS No. 127-08-2), pepsin from porcine gastric mucosa (Cat No. 1002973955), and pancreatin from porcine pancreas (4xUPS, Cat No. 1001201425) were purchased from Sigma-Aldrich. Fluorescein isothiocyanate (FITC, No. F109), sodium chloride (CAS No. 7617-14-5), Sybr Green (Ref No. S7563), and 10× phosphate buffer (PBS, Ref No. BP399-4) were acquired from Thermo Fisher Scientific. Lactobacilli MRS broth and agar were from Neogen, and magnesium nitrate (Cat No. 13446-18-9) was from Bioworld.

### Bacterial strains, growth conditions, and enumeration

*L. rhamnosus* GG strain ATCC53103 (wild type, “WT”) and its knockout mutant for the SpaCBA pilus, *L. rhamnosus* GG strain CMPG5357 (“Δ*spaCBA”*) (34), were stored at −80°C in MRS media supplemented with glycerol 25% (vol/vol). For every set of experiments, cells were recovered from the glycerol stocks by inoculating them at 10% (vol/vol) in fresh MRS broth and incubating it statically overnight at 37°C under aerobic conditions, for two consecutive days. To enumerate viable cells, bacterial suspensions were serially diluted in 1× PBS, plated in MRS agar, and incubated for 48 h at 37°C under aerobic conditions. When culturing and counting *L. rhamnosus* GG *ΔspaCBA*, the media and agar were supplemented with tetracycline (10 µL/mL) to ensure the mutant phenotype.

### Preparation of gels

Suspensions of WPI (7.5% wt/vol) and modified starch (20% wt/vol) were prepared in autoclaved distilled water and hydrated under constant stirring for 1 h at room temperature. Later, the WPI was incubated in a water bath at 90°C for 10 min and allowed to cool down back to room temperature. Both suspensions were mixed at a ratio of WPI to modified starch of 1:1 (wt/wt), the final mixture having a total solid content of 11%. Cultures of *L. rhamnosus* GG WT or Δ*spaCBA* containing 10^10^ CFU/mL were harvested on late exponential phase, centrifuged at 7,000 × *g* (Eppendorf centrifuge 5804, rotor FA-45-6-30) for 10 min, and washed twice with 1× PBS. Cell pellets were resuspended in distilled water, incorporated at 10% vol/vol into the mixture of WPI and modified starch (final cell density 10^9^ CFU/mL), vortexed, and incubated at room temperature (~23℃) under static conditions until gelling. Gels containing *L. rhamnosus* GG Δ*spaCBA* were supplemented with tetracycline (10 µL/mL) prior to inoculation. The ratio of WPI to modified starch was chosen based on the author’s empirical observation of this mixture gelled during previous work; lowering the proportion of WPI to 0.6:1 prevents the gelling from occurring (27). To enumerate viable cells, 1 g of gel was suspended in 20 mL of NaCl 0.85% (wt/vol) and homogenized using an Ultra-Turrax homogenizer T-18 (IKA Works, Inc., Wilmington, NC, USA) equipped with an S25N-18G dispersing tool, at 11,000 rpm for 1 min. The obtained suspension was used to enumerate viable cells as described above (see “Bacterial strains, growth conditions, and enumeration”).

### Acidification and turbidimetry kinetics

Kinetic studies were performed using *L. rhamnosus* GG strain WT and Δ*spaCBA*. For these, gels were obtained as described above (see “Preparation of gels”); controls were prepared by substituting the equivalent volume of WPI with distilled water or modified starch with 1× PBS buffer. For acidification kinetics, immediately after inoculation, the pH of the gels and controls was recorded every 5 min for 1 h, using a pH meter (Accumet AB15, Fisher Scientific). The acidification rate was calculated as the slope of the curve at the linear portion. Turbidimetric gelling kinetics were studied as described by Sackett et al. (37) with modifications. Briefly, 100 µL of the mixture of WPI and modified starch (or the controls) were added to 96-well plates and inoculated with the corresponding *L. rhamnosus* GG strain. Immediately, absorbance at 515 nm (28) was recorded for 1 h at room temperature (~23°C) using a SpectraMax 340 microplate reader (Molecular Devices, San Jose, CA, USA); distilled water was used as blank. The optical density (OD) is presented in its normalized form according to the following formula:


$$Normalized\;OD=\frac{OD-OD_{0}}{OD_{max}-OD_{0}}$$

Where OD is the OD at a given time, OD_0_ is the OD at 0 min, and OD_max_ is the maximum OD recorded. The acidification and turbidimetric kinetic data were plotted against each other (Fig. S1), and the inflection point leading to the exponential increase in the turbidity was taken as the pH_ɸ1_, while the inflection point when the OD stabilized was the pH_max_ (38, 39).

### Rheological measurements

Rheological measurements were made using an MCR92 Rheometer (Anton Paar GmbH, Graz, Austria) equipped with a plate-plate measuring system (50 mm diameter), a Peltier temperature controller, and managed by RheoCompass software (V1.30.1164). Gelation was studied in samples prepared with either *L. rhamnosus* GG WT or Δ*spaCBA*, by loading the mixture of WPI and modified starch after inoculation and recording the storage (G’) and loss (G”) modulus on a time-sweep at 0.3% strain, 10 Hz, 23°C, and 1 mm gap (there was a ~ 2 min delay between inoculation and the first measurement, due to the time required to load and trim the sample). The strain and frequency used for the gelation experiment fall within the viscoelastic region of both the gelled sample and the WPI/modified starch before inoculation, as confirmed via amplitude (0.001%–1%) and frequency (0.1–100 rad/s) sweeps of both types of samples at a constant 1 mm gap and 23°C (data not shown).

### Confocal imaging

Gels containing *L. rhamnosus* GG WT or Δ*spaCBA* (prepared 24 h in advance) were sliced at a thickness of 150 µm using an EMS 5000 tissue slicer (Electron Microscopy Sciences, Hatfield, PA, USA). WPI, starch, and bacterial cells within the gels were stained with FITC (10 µg/mL), Rhodamine B (500 ng/mL), and 5× Sybr green, respectively. Images were acquired with a Leica TCS SP8 STED 3× confocal microscope (UC Davis, Advanced Imaging Facility) using a 40×/1.3 oil objective and setting the excitation/emission at 500/568 for FITC and Sybr green, and 564/661 for Rhodamine B.

### Simulated gastrointestinal digestion

Digestion was performed with gels containing *L. rhamnosus* GG WT or Δ*spaCBA* (prepared 24 h in advance). For this, 1 g of gel (or the equivalent number of free cells in log basis) was suspended in 10 mL of simulated gastric fluid and incubated for 2 h at 37°C with orbital agitation at 200 rpm. Mixtures at the end of the gastric digestion were centrifuged at 7,000 × *g* for 10 min, the supernatant decanted, the pellets resuspended in 10 mL of simulated intestinal fluids and incubated for 2 h under the same conditions. Viable cells were recovered by centrifugation at 7,000 × *g* for 10 min, washed with PBS 1×, and counted (right before digestion, and at the end of the gastric and intestinal phase) as described in “Bacterial strains, growth conditions, and enumeration,” above. Experiments were performed in biological triplicates. The fluids were prepared on the day of the experiment as follows. Simulated gastric fluid (pH 3) encompassed an autoclaved solution of NaCl 0.5% wt/vol (pH adjusted with HCl 1 M), to which 3.2 mg/mL of pepsin was added right before incubation with the gels. Intestinal fluid (pH 7.5) was prepared by autoclaving a 0.025 M potassium phosphate dihydrogen buffer (pH adjusted with NaOH 1 M) with NaCl 0.5% wt/vol, and then adding 3.33 mg/mL of CaCl2, 0.5% wt/vol bile salts, and pancreatin (100 U/mL) right before incubation with the gels. Survival was calculated for each digestion phase as the percentage of viable cells at the end of the incubation relative to the cells in the gels before the digestion.

### Release of the cells from gels

One gram of gel containing *L. rhamnosus* GG WT or Δ*spaCBA* (prepared 24 h in advance) was incubated for 2 h at 37°C with orbital agitation at 200 rpm, in 10 mL of a solution at pH 3 or 7.5. Solutions were prepared as those used for the simulated digestions but without the addition of pepsin, pancreatin, or bile salts. After 2 h of incubation, a 500 µL aliquot of the supernatant was centrifuged at 7,000 × *g* for 10 min, resuspended in PBS 1×, and viable cells were counted. Then, the remaining mixture was centrifuged at the same conditions to recover all cells, washed in PBS 1×, and enumerated. The cells released from the bulk gels (those found in the supernatant before centrifugation) were expressed as a percentage of the total number of viable cells at a given time (those in the sample post-centrifugation). The experiment was performed in biological triplicates.

### Survival during storage

One gram of gels with *L. rhamnosus* GG WT was prepared on 1.7 mL centrifuge tubes and stored in stainless steel canisters kept at a constant relative humidity of 25%, 50%, or 75%. To reach the desired condition, canisters contained a saturated solution of potassium acetate, magnesium nitrate, and sodium chloride, respectively. Day zero of storage was considered to start after 1 wk when the containers reached the desired relative humidity. Viable cells were quantified (as described in “Bacterial strains, growth conditions, and enumeration,” above) every 6 days for a period of 60 d. The experiment was performed in biological triplicates for each timepoint.

### Statistical analysis

All statistical analyses were performed using RStudio (V 1.3.1093). The kinetics parameters of gels made with *L. rhamnosus* GG WT or Δ*spaCBA* were compared by a bilateral Student *t*-test at a significance level of 95%. The survival of *L. rhamnosus* GG during gastrointestinal digestion was analyzed by a two-way analysis of variance (ANOVA) to evaluate the (interactive) effect exerted by both the strain (WT vs Δ*spaCBA*) and the delivery mode (embedded in the gels or in free form). For all groups, the ANOVA was performed separately for the gastric digestion and for the gastric followed by intestinal digestion. A Tukey post hoc test at a 95% confidence interval was used to screen for pairwise differences in survival.

## RESULTS AND DISCUSSION

### *L. rhamnosus* GG induces rapid gelling of a mixture of whey protein isolate and starch via *in situ* fermentation

**Fig 1:**
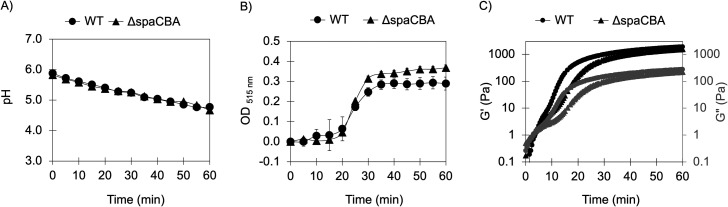
Changes in pH (**A**), optical density (**B**), and viscoelastic properties (**C**) of a mixture of WPI/modified starch after inoculation with *L. rhamnosus* GG strain WT or its pilus-depleted mutant strain Δ*spaCBA*. Rheological measurements were carried out at 10 Hz, 0.3% strain, 1 mm gap.

We evaluated the gelling of a mixture of WPI and modified starch, assembled via *in situ* fermentation by *L. rhamnosus* GG (WT strain) or its pilus-depleted mutant (Δ*spaCBA* strain). The gelling was studied based on changes in the pH (induced by microbial fermentation), optical density at 515 nm, and viscoelastic properties of the mixture as a function of time after inoculation of *L. rhamnosus* GG to the mixture (Fig. 1). Based on these measurements, kinetic parameters of the gelation process were calculated (Table 1).

**TABLE 1:** Kinetic parameters of the gelation of a mixture of WPI/modified starch via *in situ* fermentation by *L. rhamnosus* GG strains^*a*^

Kinetic parameter	*L. rhamnosus* GG strain		*P* value
	WT	Δ*spaCBA*	
Rate of acidification (units of pH drop/min)	0.024 ± 0.001	0.023 ± 0.002	0.50
pH_ɸ1_	5.49 ± 0.05	5.42 ± 0.01	0.75
Time at pH_ɸ1_ (min)	16.33 ± 2.88	19.00 ± 1.00	0.24
pH_max_	5.26 ± 0.11	5.18 ± 0.02	0.35
Time at pH_max_ (min)	29.00 ± 6.08	32.66 ± 1.15	0.40
Drop in pH after 1 h	1.11 ± 0.05	1.15 ± 0.10	0.66

^*a*^ Results are presented as the average ± standard deviation, *n* = 3.

The pH of the WPI/modified starch suspension was 5.89 ± 0.01. After inoculation, the pH decreased at a constant rate of −0.02 units/min (independent of the strain type) and reached a plateau value of 4.77 ± 0.00 and 4.67 ± 0.07 for *L. rhamnosus* GG WT and Δ*spaCBA*, respectively (Fig. 1A; Table 1). This change in pH resulted from the microbial metabolization of fermentable sugars in the modified starch suspension, as indicated by the drop in pH observed when the modified starch was inoculated in the absence of WPI but not detected when the WPI was inoculated in the absence of the modified starch (Fig. S1). Because *L. rhamnosus* GG lacks the ability to metabolize starch and maltose (40, 41), the substrate for lactic acid production could only be glucose, which was released from the starch as a consequence of the modification by acid hydrolysis and extrusion (27). The WPI/modified starch mixture initially contained ~0.2% wt/wt of reducing sugars, considering the modified starch employed had a reducing sugar content of 3.44 ± 0.22 % wt/wt (quantified by the 3,5-dinitro salicylic acid method using a glucose standard curve) (27). The slower drop in pH of the WPI/modified starch mixture compared to the modified starch suspension alone (Fig. S1) was attributed to the buffering capacity of WPI (42).

Incubating either strain of *L. rhamnosus* GG (WT or Δ*spaCBA*) with the mixture of WPI/modified starch for 1 h was sufficient for the pH to reach a steady state value of ~4.6 (Fig. S2). The lack of a further decrease could be explained by three non-mutually exclusive factors. First, *L. rhamnosus* GG WT modulates its pyruvate metabolism at low pH, resulting in a reduced production of lactate (e.g., at pH 4.8 vs 5.8) (43). Second, the final pH observed might also be related to the buffering capacity of WPI, as proteins in whey display a maximum buffering capacity at pH 4–4.7 (44), similar to the equilibrium pH in the gels obtained in this work. Third, the eventual depletion of metabolizable sugars (45) may also influence the final pH of the gels.

The rapid decrease in pH, driven by the lactic acid bacteria fermentation, caused the gelling of the WPI/modified starch suspension. Five minutes post-inoculation, the (rheological) gel point of the system was achieved as depicted by the value of G’ surpassing the G” (Fig. 1B). The gelation of the WPI/modified starch mixture was also monitored via changes in the optical density at 515 nm (OD_515nm_) as acid-induced gels are opaque while the original polymeric suspension is more translucent. Ten minutes after inoculation, the OD_515nm_ started to increase slowly, and at 20 min, an exponential increase was observed (Fig. 1D). Respectively, these changes in OD_515nm_ are suggestive of the formation of first, soluble, and then insoluble complexes between proteins and polysaccharides (38, 39). Thirty minutes post-inoculation, the OD_515nm_, G’, and G’’ had reached their maximum value.

The pH at which the optical density starts to increase exponentially and the pH at which optical density stabilizes are known as pH_ɸ1_ and pH_max_. The pH_ɸ1_ and pH_max_ were ~5.5 and 5.2, respectively, without a significant difference in the values for the gels inoculated with either strain of *L. rhamnosus* GG (WT or Δ*spaCBA*). The pH_ɸ1_ of the gel coincides with the average isoelectric point of WPI (~pI 5.5) (28), below which solutions of WPI hold a net positive surface charge. Negative surface charges have been reported for starches modified by acid hydrolysis and mechanical treatment (46), similar to the process the starch used here was subjected to. Therefore, it is possible that the gelation occurred due to electrostatic complexation, as it has been reported for WPI and modified starches at pH ranges where these biopolymers carry opposite charges (28).

Because *ex vivo* studies have shown that the pili of *L. rhamnosus* GG can cross-link with β-lactoglobulin (36), we had hypothesized a possible role of the pili on the gelation of the WPI/modified starch mixture by *L. rhamnosus* GG. However, the acidification, turbidimetric, and rheological data presented here showed no significant difference in the kinetics of gelation between gels assembled by either strain of *L. rhamnosus* GG (WT or Δ*spaCBA*). This indicates that the ability of this bacteria to assemble these gels, which depends on the fermentation of the sugars present in the mixture, is independent of the piliation of the cells. These results do not indicate that cross-linking between the pili and β-lactoglobulin is not taking place *in situ* within the gel*,* but rather that such cross-linking does not affect the kinetics of gelation of the system at the macroscopic scale studied here.

Lactic acid bacteria have been encapsulated in matrices of protein-polysaccharides that assemble under acidic conditions, mostly to form complex coacervates (not gels). For instance, *Lactiplantibacillus plantarum*, *Lacticaseibacillus paracasei*, and *Lactiplantibacillus paraplantarum* were encapsulated in WPI/gum arabic (47, 48); *L. plantarum* in WPI/κ-carrageenan (49); *L. paraplantarum* and *Lactococcus lactis* in casein/pectin (50); *L. plantarum* in gelatin/gum arabic (51); and *Bifidobacterium longum* in soy protein isolate/ɩ-carrageenan (52). In these cases, the gelation was induced by reducing the pH to 3–5.5 through dropwise addition of citric, lactic, acetic, or hydrochloric acid.

The use of fermentation to encapsulate lactic acid bacteria in gels has been performed mainly in protein suspensions, which form gels by aggregation near the protein’s isoelectric point. For example, Hellebois et al. (53) encapsulated *L. rhamnosus* GG cells into hydrogels assembled by fermentation of a mixture of sodium caseinate and whey protein isolate, adding glucose for fermentation, and trehalose and glycerol as cryoprotectants to preserve cells during and after freeze drying (53). The fermentation was carried out for 4 h at 37°C, but the onset of gelation (equivalent to the time at pH_ɸ1_ presented here) occurred over a range of time between 27 and 170 min, depending on the ratio of sodium caseinate to whey protein.

Others have used probiotic bacteria to generate gels by fermentation but from a food ingredient perspective, where the goal was to produce gels and not to encapsulate the bacteria *per se*. This includes the use of *L. rhamnosus*, *L. plantarum, L. lactis, Lacticaseibacillus casei, Lactobacillus delbrueckii* subsp. *bulgaricus* as well as other lactic acid bacteria to generate gels made of whey protein isolate, soy protein isolate, alginate/caseinate, or soy milk (54–57). The rheological gel points of these gels ranged between 51 and 220 min, and the final pH from 4.6 to 6.3, depending on the bacterial strain, inoculum size, and the biopolymers used (54–56). Although these gel points (defined as the time at which G’ surpasses G”) seem to take a longer time than the results in this study, it must be considered that the objective of these studies was not to rapidly encapsulate the bacteria but to produce gels. Moreover, the direct comparison among these studies is challenging due to differences in the angular frequency values selected for the analysis of rheological point gel points. Thus, while rheological data provide important information about the mechanical properties of the gels, turbidimetric kinetics might be an easier method to compare gelation times between similar systems.

### Gel microstructure and bacterial localization

**Fig 2:**
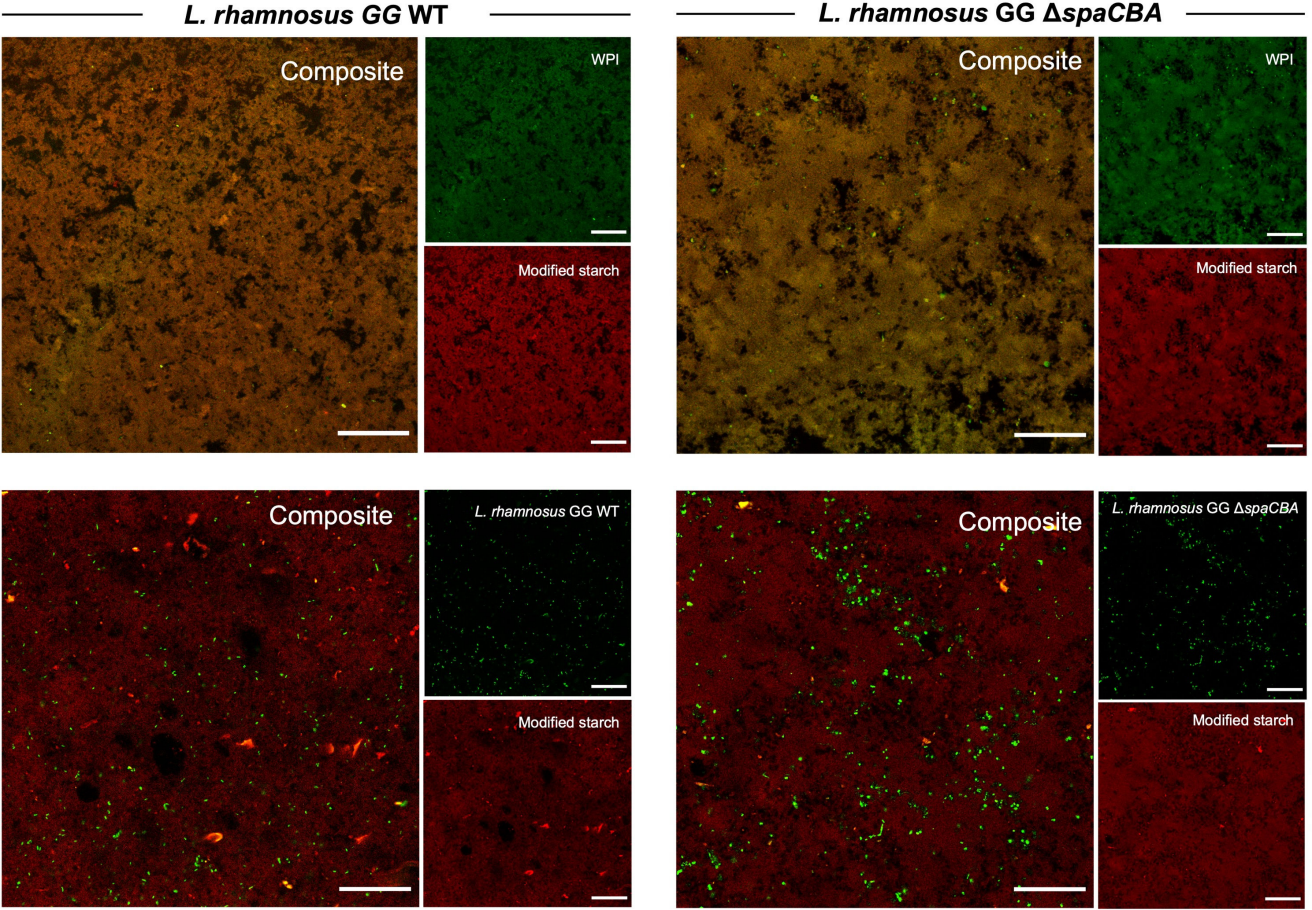
Confocal images of mixed WPI/modified starch gels assembled via *in situ* fermentation by *L. rhamnosus* GG strain WT or its pilus-depleted mutant strain Δ*spaCBA. S*cale bar, 50 µm. WPI stained with FITC (green), modified starch stained with Rhodamine B (red), and bacterial cells stained with Sybr Green (green).

Confocal images of the gels (Fig. 2) show the WPI stained with FITC (green color), modified starch with Rhodamine B (red color), bacterial cells with Sybr Green (green color), and dark zones that represent areas devoid of biopolymers or cells. Samples were co-stained with FITC and Rhodamine B to observe the microstructure of the gel and the spatial distribution of the WPI and modified starch, or with Rhodamine B and Sybr Green to elucidate the localization of the cells within the gel matrix.

Composite images of gels co-stained with FITC and Rhodamine B revealed a matrix in which the WPI and modified starch were spatially superimposed and homogeneously distributed, as depicted by the yellow color that results from the overlap of the green and red pseudo-colored fluorescent signals. Together with the acidification and turbidimetric kinetic data, this overlapped signal—which suggests the WPI and modified starch are in close physical proximity—could indicate that the gel is cross-linked via associative electrostatic interactions between the protein and polysaccharide, driven by opposite surface charges, as described by Le and Turgeon (39).

Regarding the localization of the bacterium, cells of *L. rhamnosus* GG WT appeared uniformly distributed through the gel matrix, whereas the Δ*spaCBA* mutant cells aggregated in the gel cavities (i.e., the dark zones devoid of biopolymers). While more quantitative analysis would be necessary to characterize these putative patterns, others have observed that the environment and the bacterial surface molecules impact the distribution of the cells on a given matrix. For instance, similar to what is presented here, Tarazanova et al. (58) reported that aggregated *L. lactis* cells tightly filled the cavities of the protein matrix in a fermented milk (58). Meanwhile, *L. rhamnosus* GG WT cells inoculated in raw milk preferentially formed aggregates, whereas the pilus-depleted mutant Δ*spaCBA* strain was homogenously distributed (59). Although the latter seems opposite to what was observed here (where the mutant cells aggregated but the WT were homogeneously distributed), these findings are not necessarily contradictory. Preferential bacteria-to-bacteria vs bacteria-to-matrix interactions, which govern distinct distribution patterns, are context dependent and might vary according to the matrix, pH, cell density, etc. Thus, one possible interpretation of our results is that *L. rhamnosus* GG WT cells were homogeneously distributed because they were able to adhere to the gel matrix via the SpaCBA pili, which is known to bind to whey proteins (preferentially β-lactoglobulin [33, 60]). Conversely, the Δ*spaCBA* cells might have preferentially aggregated in the gel cavities because the surface of these cells is more hydrophobic than the WT cells (32), and thus they might have been excluded from the hydrated polymer-rich areas of the hydrogel. In addition to physicochemical interactions based on surface hydrophobicity, other molecular features on the cell surface, such as the expression of exopolysaccharides and other adhesins of the Δ*spaCBA* mutant cells could promote cell–cell interactions and result in the observed aggregation behavior of these cells in the absence of the pili (30, 32).

### Protection against gastrointestinal conditions provided by the gel partially depends upon the piliation of *L. rhamnosus* GG

**Fig 3:**
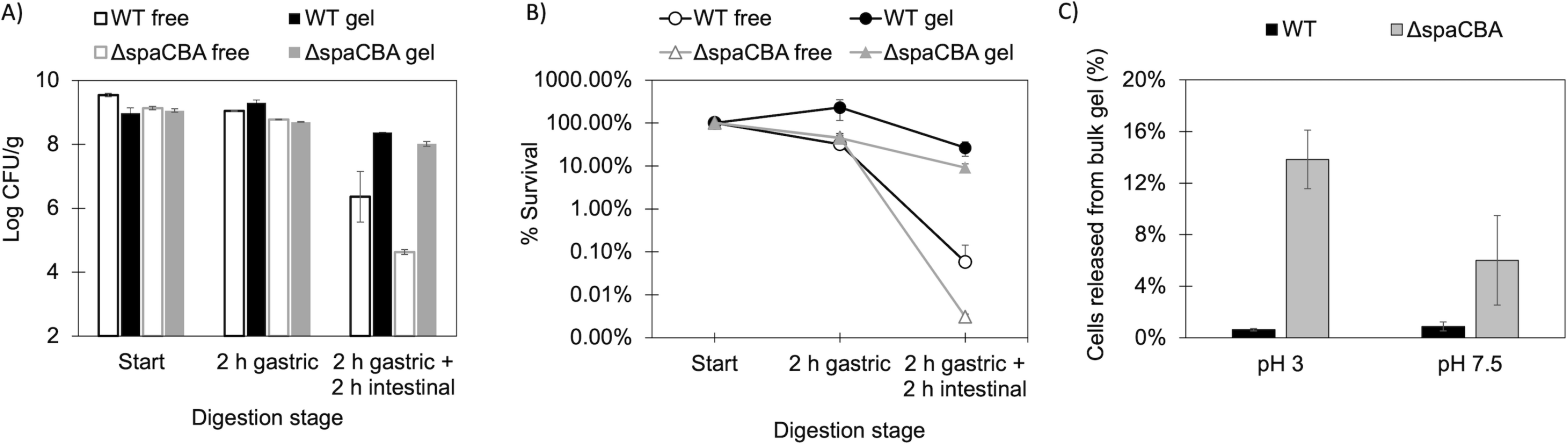
Effect of the strain (WT or Δ*spaCBA*) and delivery mode (free or encapsulated in WPI/modified starch gel) in the survival of *L. rhamnosus* GG, reported in log CFU/g (**A**) and percentage (**B**). Effect of the strain (WT or Δ*spaCBA*) and pH on the release of *L. rhamnosus* GG cells from the bulk WPI/modified starch gels (**C**).

We performed simulated gastrointestinal digestion to evaluate the extent of protection that the WPI/modified starch gel provides to *L. rhamnosus* GG cells (Fig. 3A and B). For this, gels containing ~9 log CFU/g of *L. rhamnosus* GG WT or Δ*spaCBA*, or equivalent numbers (in log basis) of free cells, were subsequently incubated for 2 h in artificial gastric (pH 3) and for 2 h in intestinal (pH 7.5) fluid. At the end of the simulated gastrointestinal digestion, counts of *L. rhamnosus* GG WT encapsulated in the WPI/modified starch gels were reduced by 0.59 ± 0.15 log CFU/g, while free cell count was reduced by 3.64 ± 0.74 log CFU/g. The significantly higher survival of WT embedded in the gels compared to free form (*P* value 7 × 10^−4^) indicated that the gel matrix protected the cells against such conditions. The protection provided by the WPI/modified starch gel might arise from the ability of WPI to buffer the pH of the media, and more importantly, the physical entrapment of the cells in the protein/polysaccharide aggregates (61). However, at the end of the digestion, the population of *L. rhamnosus* GG Δ*spaCBA* encapsulated in the gels dropped by 1.04 ± 0.04 log CFU/g, thus showing a significantly lower survival than the WT strain encapsulated in the same gels (*P* value 9 × 10^−3^). This suggests that the encapsulation within the gel, on its own, is insufficient to fully explain its protective effect.

We hypothesized that the protection provided by the gel could partially depend upon the physical attachment of the bacteria to the gel matrix mediated by the SpaCBA pili. Thus, we investigated the release of *L. rhamnosus* GG cell strains (WT or Δ*spaCBA*) from the gels (Fig. 3C). For this, the gels were separately incubated for 2 h in solutions such as those used for the simulated digestion but prepared without the addition of the pepsin, bile salts, or pancreatin (to minimize the loss of cell viability). We then calculated the percentage of cells released from the bulk gels (which remain at the bottom of the tubes) by determining the ratio of cells in the supernatant (i.e., released cells) vs in the precipitate post-centrifugation (i.e., total cells). After 2 h of incubation at pH 3 or 7.5, only 0.66% ± 0.09% and 0.94% ± 0.37% of the *L. rhamnosus* GG WT cells, respectively, were released from the bulk gels. In contrast, 6.32% ± 3.65% and 14.57% ± 2.01% of *L. rhamnosus* GG Δ*spaCBA* were released. We interpret these results, combined with the patterns observed in the confocal micrographs, as an indication that the pili of *L. rhamnosus* GG promote a higher retention of the cells within the gel matrix via physical bacteria-to-matrix interactions. This would explain why, in the absence of the pili, the encapsulated mutant Δ*spaCBA* had a lower survival than the encapsulated WT strain under simulated gastrointestinal conditions. Burgain et al. (31) demonstrated that the SpaCBA pilus of *L. rhamnosus* GG was crucial to retain the bacterium during their encapsulation in a matrix made of a mixture of micellar casein and WPI, obtained by a patented method involving emulsification followed by freeze drying (31). Based on their results, the authors advised that understanding possible interactions between probiotic bacteria and their surrounding encapsulation matrix was key to yielding high encapsulation efficiencies. Here, our data open the possibility that this idea extends beyond the process of encapsulation, as the physical interactions between *L. rhamnosus* GG and the gel matrix might impact the protection it provides to the cells upon their passage through the gastrointestinal tract. However, *in vivo* studies should validate the relevance and mechanism by which these bacteria–matrix interactions might impact the delivery of probiotics. One possible mechanism is the physical retention of the cells within the encapsulation matrix, as suggested by our *in vitro* data. Other factors might include changes in bacterial phenotype caused by the attachment of the cells to the matrix. For example, *L. rhamnosus* GG is also capable of adhesive interaction with milk fat globule membrane via the SpaCBA pili (62). This binding interaction has been shown to change the expression of *L. rhamnosus* GG genes related to exopolysaccharide production and to result in increased survival of the bacteria in the cecum of mice (63).

The survival of free WT and Δ*spaCBA* cells at the end of the *in vitro* digestion was statistically the same (*P* value 0.99), suggesting these strains have a similar tolerance to the simulated gastrointestinal conditions, including acid and bile stress. These results agree with the work of Ardita et al. (64) that reported equivalent counts of *L. rhamnosus* GG WT or its pilus mutant (Ω*spaC*::Eryr, CMPG10102) in the unwashed jejunum of mice previously administered with a single dose of either strain in free form (64).

### Hydrated gel protects *L. rhamnosus* GG during unrefrigerated storage

**Fig 4:**
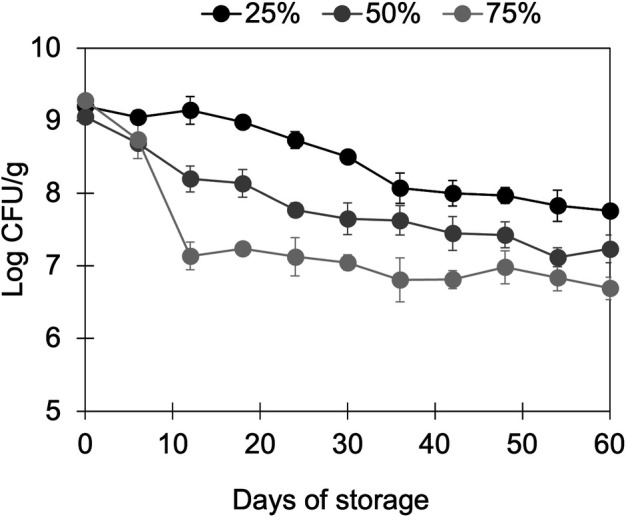
Effect of relative humidity (%) on the viability of *L. rhamnosus* GG WT encapsulated in WPI/modified gels during room temperature storage.

Lastly, we evaluated the viability of *L. rhamnosus* GG WT encapsulated in the WPI/modified starch gels (initially 9.18 ± 0.12 CFU/g) during storage at room temperature (Fig. 4). Room temperature was selected because this would reduce the cost of storing the product, in alignment with the perspective of developing encapsulation technologies that could be deployed in low-income settings. Moreover, we chose to perform this experiment with the *L. rhamnosus* GG WT strain only because that is the probiotic strain of interest, and it showed the highest survival under gastrointestinal conditions when encapsulated in the gels. After 60 days of storage at relative humidity of 25%, 50%, or 75%, the final cell counts were 7.76 ± 0.06, 7.24 ± 0.19, and 6.69 ± 0.15, respectively. As expected, a higher loss of cell viability was observed with increasing relative humidity (65).

Remarkably, in this work, the gels were stored at room temperature in their hydrated state (~89% water content), meaning without needing any drying or refrigeration. In contrast, others have shown comparable or higher losses of viability for lactic acid bacteria encapsulated in dried matrices made of mixed protein and polysaccharides with moisture contents ranging from 3% to 10%. For example, a freeze-dried coacervate of WPI/gum arabic (pH 3.75) with embedded *L. plantarum* showed reductions in the viability of 1.5–2.5 log CFU/g after 60 days of storage at room temperature (48), while the same species of bacteria, but encapsulated in a spray-dried gelatin/gum arabic coacervate (pH 5.5), displayed a reduction of 6 log after 20 days of storage at a relative humidity of 33% (51). *B. longum* in freeze-dried soy protein isolate/ɩ-carrageenan coacervates (pH 3.75) lost 1–2 log CFU/mL after 30 days of storage at 4°C, depending on the ratio of protein/polysaccharide (52). In part, powder formulations aim to reduce the cost of refrigerated storage or transportation often associated with probiotic products, but the drying processes used to obtain these formulations are either expensive, such as freeze drying, or might negatively impact cell viability and metabolism, as spray drying might (66). Therefore, the WPI/modified starch (hydrated) gels might provide a sustainable alternative to other more expensive or energy-intensive encapsulation systems for the stabilization of *L. rhamnosus* GG during unrefrigerated storage.

Another consideration when using mixtures of protein-polysaccharide structured through electrostatic interactions (whether they form gels or complex coacervates) for the purpose of microbial encapsulation is that the optimal pH for the interaction between the polymers should be compatible with the survival of the cells during storage. In the gel presented here, the final pH was ~4.6, a value typically found in fermented foods from which lactic acid bacteria are well adapted. *L. rhamnosus* GG is able to withstand this condition by upregulating the expression of genes such as that of F_0_F_1_-ATPase (an enzyme that pumps protons out of the cells upon hydrolysis of ATP) and slowing down its own production of L-lactate by reducing the production of L-lactate dehydrogenase (43). Beyond being compatible with cell viability, one can speculate that the final pH of the encapsulation systems could be tuned (within the range at which the protein/polysaccharides interact to form encapsulation matrices) to promote more resistant phenotypes on the embedded cells. For instance, incubating free *L. rhamnosus* GG WT cells at pH 4.5 for 1 or 24 h resulted in increased *in vitro* binding of the bacteria to mucin and in higher survival of the cells in the intestinal tract of mice (compared to non-acid stressed cells) (67). Thus, future work might explore the effect that the pH of protein-polysaccharide gels has on probiotic storage and delivery. For this, precise control of the final pH of the gels could be achieved by adjusting the concentration of the inoculum and the metabolizable sugars in the polymeric mixture (57), an approach compatible with food manufacturing practices in the yogurt and cheese processing industries. However, the fact that cellular responses to pH are strain specific warrants further investigation into the applicability of this approach for different probiotic strains.

### Conclusion

This work demonstrated that *L. rhamnosus* GG cells rapidly encapsulate themselves in a gel assembled by *in situ* fermentation of a mixture of WPI and modified starch. This process relied on the acidifying nature of this lactic acid bacterium, provided with metabolizable sugars found in the modified starch suspension. The pili of *L. rhamnosus* GG had no effect on the gelation kinetics or in the survival of the free cells during simulated digestion *per se*. However, the piliation of the cells seems to impact the localization of the bacterium within the gel and to promote their retention in such a matrix, which could partially explain the protection that the gel provided to the encapsulated bacteria during simulated digestion. *In vivo* studies may be designed in the future to validate the relevance of such bacteria-to-matrix interactions in the survival of the cells through digestion. Lastly, the hydrated gels sustained viable counts of *L. rhamnosus* GG up to 7.7–6.7 log CFU/g after 60 days of storage at room temperature, depending on the relative humidity. Thus, the WPI/modified starch gels might be suitable to preserve the cells during unrefrigerated short-term storage, without the need for drying or refrigeration. Further research may evaluate the role that bacteria-to-matrix interactions or the metabolites produced during fermentation play in the observed stability of the encapsulated *L. rhamnosus* GG during storage, and the applicability of this approach for other probiotic strains.

## SUPPLEMENTAL MATERIAL

Fig. S1 and S2 aem.00248-25-s0001.docx Changes in pH and optical density of polymeric solution inoculated with LGG and its pilus-depleted mutant.

ASM does not own the copyrights to Supplemental Material that may be linked to, or accessed through, an article. The authors have granted ASM a non-exclusive, world-wide license to publish the Supplemental Material files. Please contact the corresponding author directly for reuse.
