# Supplementary material for: Influence of the pili of Lacticaseibacillus rhamnosus GG on its encapsulation and survival in mixed protein-starch gels assembled by in situ fermentation
Source: Appl Environ Microbiol. 2025 Jun 12;91(7):e00248-25. doi: 10.1128/aem.00248-25 (PMC12285250; doi:10.1128/aem.00248-25)
Supplement: Fig. S1 and S2 — Changes in pH and optical density of polymeric solution inoculated with LGG and its pilus-depleted mutant. [file aem.00248-25-s0001.docx]

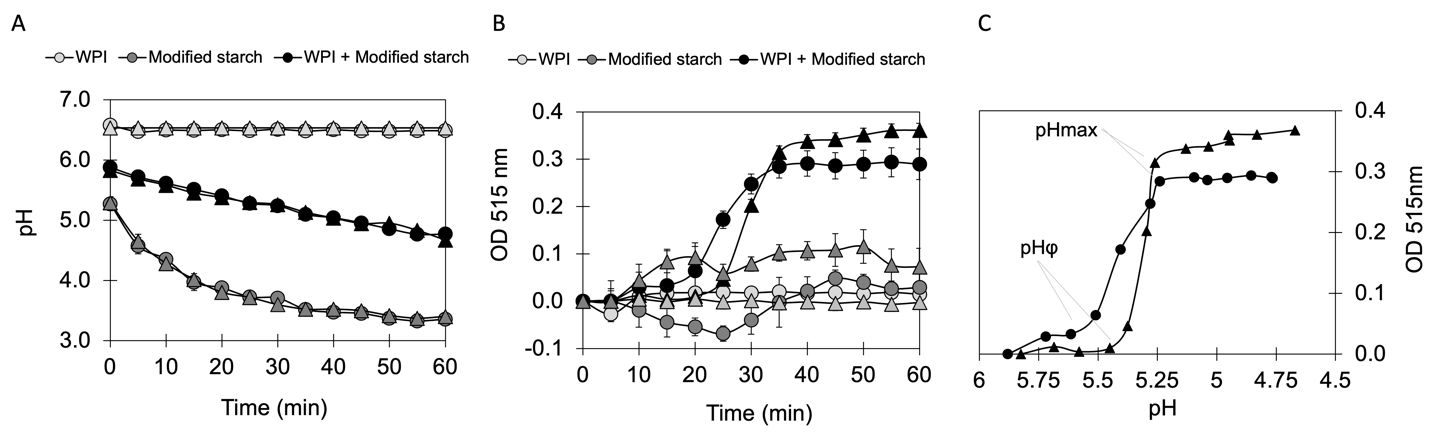


**Supplementary Figure 1**. Changes in pH (A) and optical density (B) of WPI, modified starch, and WPI + modified starch inoculated with *L. rhamnosus* GG strain WT (●) and Δ*spaCBA* (▲). The optical density was plotted as a function of pH for the WPI + modified starch mixture inoculated with either strain (C).


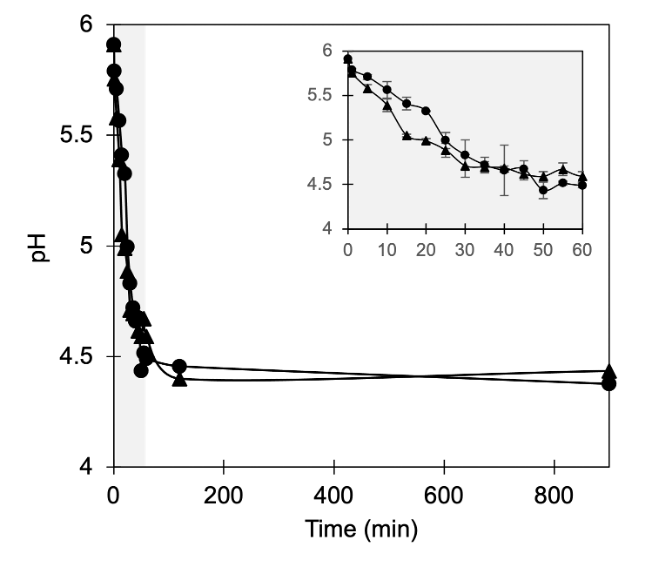


**Supplementary Figure 2.** Change in pH of a mixture of WPI and modified starch due to fermentation by *L rhamnosus* GG strain WT (●) or its pilus-depleted mutant strain ΔspaCBA (▲).
